# The Concentrations of Ambient *Burkholderia Pseudomallei* during Typhoon Season in Endemic Area of Melioidosis in Taiwan

**DOI:** 10.1371/journal.pntd.0002877

**Published:** 2014-05-29

**Authors:** Ya-Lei Chen, Yu-Chuan Yen, Chun-Yuh Yang, Min Sheng Lee, Chi-Kung Ho, Kristina D. Mena, Peng-Yau Wang, Pei-Shih Chen

**Affiliations:** 1 Department of Biotechnology, National Kaohsiung Normal University, Kaohsiung, Taiwan; 2 Department of Public Health, College of Health Science, Kaohsiung Medical University, Kaohsiung, Taiwan; 3 Department of Pediatrics, Kaohsiung Medical University Hospital, Kaohsiung Medical University, Kaohsiung, Taiwan; 4 Epidemiology, Human Genetics, and Environmental Sciences, School of Public Health, University of Texas Health Science Center at Houston, Houston, Texas, United States of America; 5 Disaster Prevention & Water Environment Research Center, National Chiao Tung University, Hsin Chu, Taiwan; 6 Institute of Environmental Engineering, College of Engineering, National Sun Yat-Sen University, Kaohsiung, Taiwan; Beijing Institute of Microbiology and Epidemiology, China

## Abstract

**Background:**

Melioidosis is a severe bacterial infection caused by *Burkholderia pseudomallei* with a high case-fatality rate. Epidemiological and animal studies show the possibility of inhalation transmission. However, no *B. pseudomallei* concentrations in ambient air have been researched. Here, we developed a method to quantify ambient *B. pseudomallei* and then measured concentrations of ambient *B. pseudomallei* during the typhoon season and the non-typhoon season to determine the factors influencing ambient *B. pseudomallei* levels.

**Methods:**

We quantified ambient *B. pseudomallei* by using a filter/real-time qPCR method in the Zoynan Region in Kaohsiung, southern Taiwan. Twenty-four hour samples were collected at a sampling rate of 20 L/min every day from June 11 to December 21, 2012 including during the typhoon season (June to September) and reference season (October to December).

**Results:**

We successfully developed a filtration/real-time qPCR method to quantify ambient *B. pseudomallei*. To our knowledge, this is the first report describing concentrations of ambient *B. pseudomallei*. Ambient *B. pseudomallei* were only detected during the typhoon season when compared to the reference season. For the typhoons affecting the Zoynan Region, the positive rates of ambient *B. pseudomallei* were very high at 80% to 100%. During June to December, rainfall was positively correlated with ambient *B. pseudomallei* with a statistical significance. Sediment at a nearby pond significantly influenced the concentration of ambient *B. pseudomallei*. During the typhoon month, the typhoon was positively correlated with ambient *B. pseudomallei* whereas wind speed was reversely correlated with ambient *B. pseudomallei*.

**Conclusions:**

Our data suggest the possibility of transmission of *B. pseudomallei* via inhalation during the typhoon season.

## Author Summary

Melioidosis is a severe bacterial infection caused by *Burkholderia pseudomallei* with a high case-fatality rate. Epidemiological and animal studies show the possibility of inhalation transmission. However, no *B. pseudomallei* concentrations in ambient air have been researched. Here, we successfully developed a method to quantify ambient *B. pseudomallei* by using a filter/real-time qPCR method. Twenty-four hour samples were collected every day from June 11 to December 21, 2012 including during the typhoon season (June to September) and reference season (October to December) in the Zoynan Region in Kaohsiung, southern Taiwan. To our knowledge, this is the first report describing concentrations of *B. pseudomallei* in ambient air. For the typhoons affecting the Zoynan Region, the positive rates of ambient *B. pseudomallei* were very high. Our data imply the possibility of air transmission of Melioidosis during the typhoon season. In addition, ambient *B. pseudomallei* aerosolized from sediment of a nearby lake should be a concern as an important source of transmission. Our results could provide deeper insight into Melioidosis transmissibility and infection control.

## Introduction

Melioidosis, which is endemic in northern Australia and Southeast Asia, is an emerging infection in other Asian regions and in South America [1]–[6]. Sporadic autochthonous cases have also been reported throughout the world, including Africa, the Caribbean, America, and the Middle East [1], [7]–[8]. Melioidosis is a severe bacterial infection caused by *Burkholderia pseudomallei* with high case-fatality rates of 14% to 40% in Thailand, Australia, Singapore and Taiwan [1], [6], [9]–[11]. With increasing worldwide travel of both humans and animals, and the concern of *B. pseudomallei* as an important potential bioweapon, this agent is an emerging global public health problem [1]–[3], [7], [10], [12].

Risk factors of melioidosis are diabetes, hazardous alcohol use, chronic lung or renal disease, and older age [1], [10], [13]. Pneumonia is the most common clinical presentation [1], [5], [10], [14]–[15]. The association between melioidosis and rainfall intensity is well documented from endemic regions [5]–[6], [10], [13]–[14]. A significant linear correlation has been observed between rainfall and melioidosis cases [1], [5]–[6], [13], [15]–[16]. The case clusters were also associated with sudden and heavy rainfall related to cyclones and typhoons [1], [4], [6], [10], [14]–[18].

Although outbreaks have been linked to contamination of drinking water [1], it is now believed that percutaneous inoculation is the major mode of acquisition [1], [19]–[21]. Recently, epidemiological studies support inhalation as the mode of transmission of *B. pseudomallei* after heavy monsoonal rains and winds [5], [6], [10], [12]–[13], [17], [21]–[22]. Animal studies also show that exhaled *B. pseudomallei* aerosols lead to a lower lethal dose, 50% (LD_50_) and a shorter incubation time compared to intraperitoneal and subcutaneous injection [23]–. However, no *B. pseudomallei* concentrations in ambient air have been researched.

There are very limited researches regarding quantifying ambient pathogens. The first study of quantifying airborne pathogens by using filter/real-time qPCR was to determine the concentrations of airborne *M. tuberculosis* in hospitals [25]–[26]. Then, airborne influenza virus and avian influenza virus was also quantified in poultry markets [27]. Recently, the first report describing the concentration of ambient pathogens implied the possibility of long-range transport of influenza virus because the concentration of ambient influenza A virus was significantly higher during the Asian dust storm days than during the background days [28]. This quantitative method shows promise for quantifying ambient pathogens with high sensitivity and specificity and should provide deeper insight into infectious disease transmissibility and epidemiology, as well as infection control. Therefore, the aim of the current study was to develop a method to quantify ambient *B. pseudomallei* and to determine the factors influencing ambient *B. pseudomallei* levels.

## Materials and Methods

### Reference bacteria and genomic DNA

Reference strains of *B. pseudomallei* (vgh19) were kindly provided by the laboratory for Biotechnology, National Kaohsiung Normal University (Kaohsiung, Taiwan); their characteristics have been described in previous studies [29]. We target the *B. pseudomallei* – specific type III secretion system (TTSS) gene cluster encompassing part of open reading frame 2 (orf2) [30], which was recently reported to be the most accurate clinically and has shown sensitivity beyond culture on soil samples for *B. pseudomallei* detection [31]. The target DNA standard solution of *B. pseudomallei* was purchased from Mission Biotech (Taipei, Taiwan).

### DNA isolation and quantification

We extracted genomic DNA from *B. pseudomallei* with the QIAamp DNA Mini kits (QIAGEN, GmbH, Hilden, Germany) as previously described [26]. The bacterial DNA was stored at −20°C within one month before the analysis.

For *B. pseudomallei*, the primers and probe were primarily targeted to TTSS-*orf*2 with the sensitivity and specificity of 100% for *B. pseudomallei* [30]. The sequence of primers were BpTT4176F (5′-CGTCTCTATACTGTCGAGCAATCG-3′), BpTT4290R (5′-CGTGCACACCGGTCAGTATC-3′), and the probe was fluorogenic probe BpTT4208P (5′-CCGGAATCTGGATCACCACCACTTTCC-3′). The PCR assay was performed at a final volume of 25 µl and 45 cycles as previously described [32] and amplification and detection were performed on the 7900HT Fast Real-Time qPCR System (Applied Biosystems, Inc., Foster City, CA).

Ten-fold serial dilutions of the target DNA standard solution was made for the calibration curve. In order to achieve PCR efficiency of 90% to 110%, the slope of the calibration curve must range from −3.6 to −3.1. The R^2^-value must be above 0.99. The positive and negative controls were analyzed with each run. The negative controls were all uncontaminated. All samples, positive controls and negative controls were analyzed in triplicate.

### Chamber study

#### Comparison of real-time qPCR and culture

To understand the relationship between culture and the quantitative analysis of real-time PCR, we activated the bacteria (vgh19) and cultured 100 µl to Ashdown's agar for two days. We also took 50 µl of the same batch to autoclaved polytetrafluoroethylene (PTFE; Teflon) membrane filters, then extracted DNA and detected the concentrations by real-time qPCR.

#### Comparison of filters

We spiked the same quantity of *B. pseudomallei* into 37 mm diameter with 0.1 µm pore size Teflon filter and polycarbonate (PC) filter. We compared the Teflon filter and PC filter in the laboratory after 24 hours and 48 hours sampling at 20 L/min.

#### Transport temperature

In order to determine the optimal transport temperature for the air samples, we spiked equal volumes of *B. pseudomallei* on autoclaved Teflon filters and stored at 4°C and 25°C for 24 hours and 48 hours, respectively.

#### Matrix effect

We collected air samples over 24 hours on Teflon filters at 20 L/min and then spiked with *B. pseudomallei*. In addition, we also spiked equal volumes on clean Teflon filters. Finally, we extracted DNA of *B. pseudomallei* to determine the inhibitory effect of air matrix.

### Field study

#### Sampling strategy

From 2005 to 2011, the number of melioidosis cases in Kaohsiung accounted for 70.3% cases in Taiwan and there was a hotspot of both cases and deaths in the Zoynan Region [11], [16], [22]. We chose the second floor of the elderly activity center with a distance of 11.4 meters to the Lotus Pond in the Zoynan Region in Kaohsiung, southern Taiwan, as our sampling site. Airborne *B. pseudomallei* was collected on Teflon filters in the disposable plastic cassettes by using a sampling pump operating at 20 L/m with a sampling time of 24 hours. We sampled air every day from June 11 to December 21, 2012 including the typhoon season (June to September) and the reference season (October to December). Before sampling, the filters and support pads were autoclaved, and the plastic cassettes were sterilized with ethylene oxide.

#### Environmental parameters

During the study period, the air pollutants and climate factors were provided by the Taiwan Environmental Protection Administration and the Taiwan Central Weather Bureau, respectively. The environmental parameters included concentrations of ozone (O_3_), sulfur dioxide (SO_2_), carbon monoxide (CO), and nitrogen oxide (NO_X_). The climate factors included typhoon information, rainfall, wind speed, and ultraviolet index (UV index; 1 unit equals 25 mW/m^2^). From these data we calculated the mean air pollutants and climate factors for defined periods before date of ambient *B. pseudomallei* measurement. Since there is no study regarding the appropriate day-lag of ambient pollutant concentrations and climate factors to consider when studying their relations with ambient *B. pseudomallei*, we examined the effects of 0-, 1-, 2-, 3-day lags (lag 0, lag 1, lag 2, and lag 3) and 2-days, 3-days, and 4-days sum before the date ambient *B. pseudomallei* measurement.

#### Ambient bacteria and fungi

Duplicate total cultivable airborne bacteria and fungi samples were collected using portable microbiological air sampler (MAS-100; MERCK, USA). The tryptic soy agar (TSA, Difco Laboratories, Michigan, USA) and malt extract agar (MEA, Difco Laboratories, Michigan, USA) was used for bacteria and fungi, respectively. Bacteria were incubated at 37°C for 24 hours and fungi were incubated at 25°C for 48 hours. The results were expressed as colony forming unit per cubic meter air (CFU/m^3^).

### Statistical methods

Statistical analyses were performed using SPSS for Windows Chinese Traditional 14.0. The descriptive statistics were used to evaluate the range, mean and standard error of parameters. The Mann-Whitney U test was used to evaluate the difference of ambient *B. pseudomallei* and environmental factors between different groups. Since the distribution of ambient *B. pseudomallei* did not fit to normal distribution (significant result was found in Kolmogorov-Smironov test), we used Spearman correlation to evaluate relationships between *B. pseudomallei* and meteorological factors. Significance was accepted at *p*-value<0.05.

## Results

### Development of filter/real-time qPCR method

**Figure 1:**
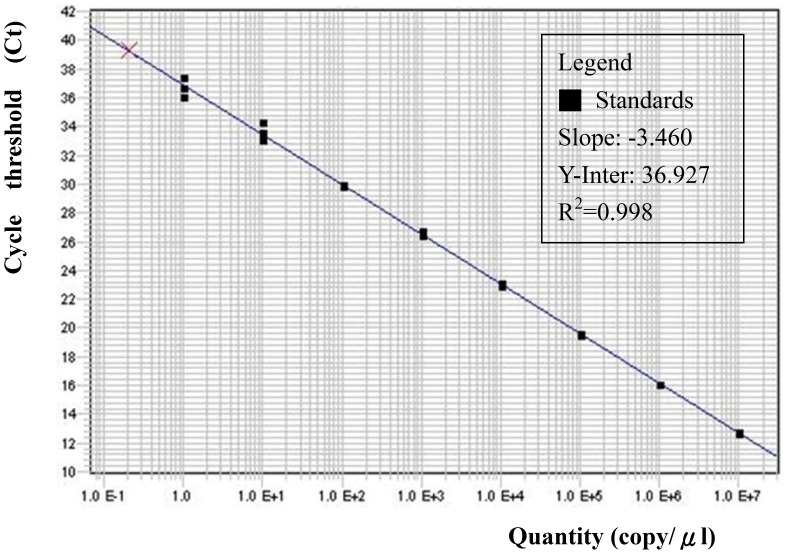
Standard curve of known DNA (1.0×10^0^ to 1.0×10^7^ copies/µl) and threshold cycle (C*t*) measured using a real-time qPCR for *B. pseudomallei* with R^2^ of 0.998 and slope of −3.46.

Figure 1 shows the calibration curve of *B. pseudomallei* from 1.0×10^0^ to 1.0×10^7^ copies/µl with R^2^ of 0.998 and slope of −3.46. Because the DNA on each filter was extracted to a final volume of 200 µl, the detection limit of the filter/real-time qPCR method for *B. pseudomallei* was 6.94 copies/m^3^. If the positive results were found in the 1/100 dilution samples due to the inhibitory effect, then the actual detection limit corrected for this effect was 0.07 copies/m^3^. Based on our results, 1 copy/µl in the real-time qPCR assay was approximately equal to 5.5 CFU/µl.

**Figure 2:**
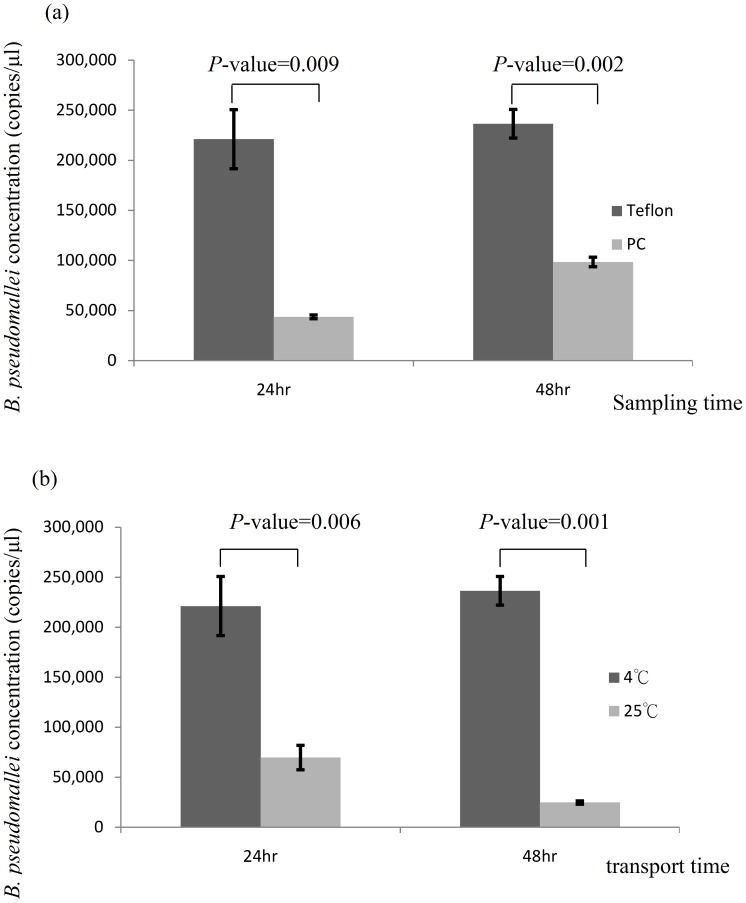
(a) Teflon and PC filters comparison over 24 hours and 48 hours sampling after spiking the same quantity of *B. pseudomallei* (b) transport temperature comparison of 4°C and 25°C over 24 hours and 48 hours after spiking the same quantity of *B. pseudomallei* on Teflon filters.

Figure 2 (a) shows the *B. pseudomallei* concentrations on Teflon and PC filters over 24 hours and 48 hours of sampling after spiking the same quantity of *B. pseudomallei*. The concentrations of *B. pseudomallei* on Teflon filters of both 24-hour and 48-hour samples were significantly higher than that on PC filters. So, we evaluated the influence of transport temperature on Teflon filters. Figure 2 (b) shows *B. pseudomallei* concentrations with the transport temperature at 4°C and 25°C over 24 hours and 48 hours for the spiked samples on Teflon filters. The concentrations of *B. pseudomallei* at 4°C for both 24-hour and 48-hour samples were significantly higher than that at 25°C. For inhibitory effect evaluation, our results show that the extracted DNA of *B. pseudomallei* on filters containing ambient aerosols (9.2×10^1^ copies/µl) were significantly lower than that on blank filters (2.1×10^2^ copies/µl) after spiking same quantity of *B. pseudomallei* (*p*-value = 0.01).

### Ambient *B. pseudomallei* and typhoon events

**Figure 3:**
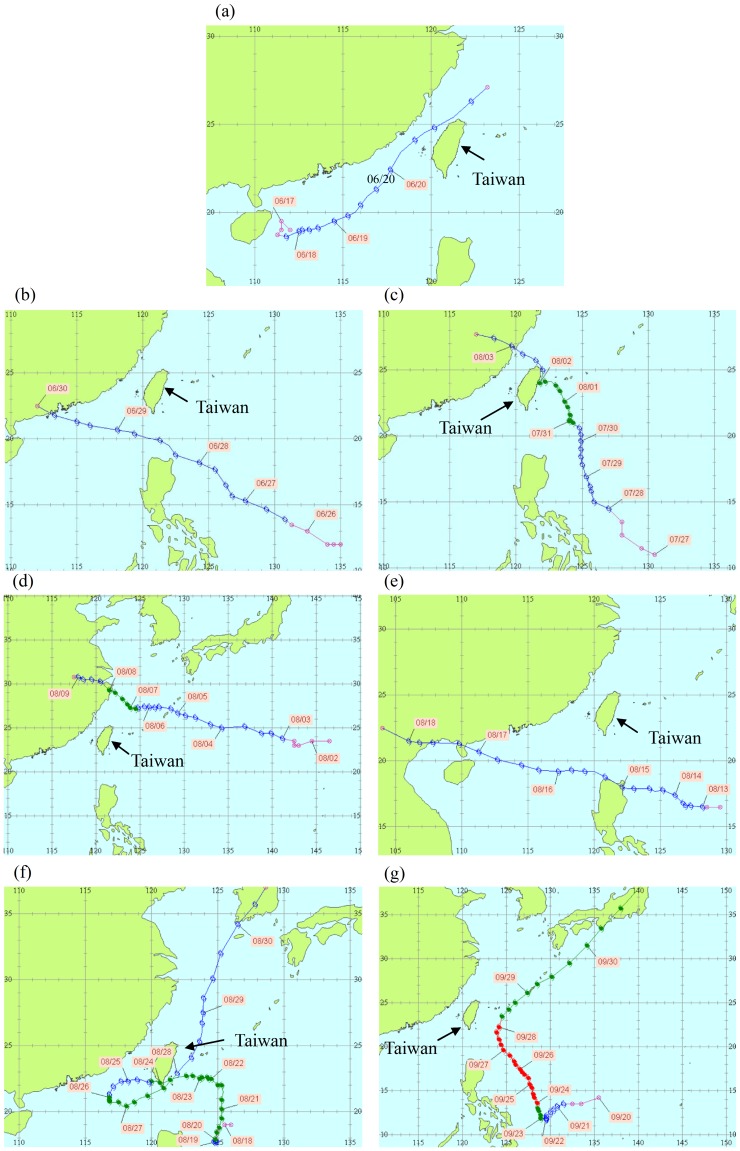
The path of (a) Talim, (b) Doksuri, (c) Saola, (d) Haikui, (e) Kai-Tak, (f) Tembin and (g) Jelawat Typhoon from Taiwan Central Weather Bureau.

We measured ambient *B. pseudomallei* from June 11 to December 31, with a total of 188 samples (we lost six samples from June 28 to June 30 and August 23 to 26). The positive rate by qPCR (the number of positive samples divided by the number of all samples) is 20.2% with the concentration range from not detected to 4×10^4^ copies/m^3^. According to Taiwan Central Weather Bureau, seven typhoons affected Taiwan in 2012 with the definition of “affected periods” as the period of issuing a sea alert to the typhoon [33]. Table 1 shows the information of the seven typhoons as well as ambient *B. pseudomallei* and environmental characteristics during the typhoon-affected periods. The positive rate during Kai-Tak and Tembin affected periods was higher than that of other typhoons. The path of Talim, Doksuri, Haikui, Kai-Tak, and Jelawat were far away from Taiwan, whereas Saola and Tembin landed Hualien (East Taiwan), and Pingtung (South Taiwan), respectively (Figure 3). Only Doksuri, Kai-Tak, and Tembin affected the southern part of Taiwan where we sampled ambient *B. pseudomallei*.

**Table 1:** Ambient *B. pseudomallei* and environmental characteristics during typhoon-affecting-period in 2012 in Taiwan.

Typhoon	The period of issuing sea alert	Characteristics		*B. pseudomallei*	
		Minimum pressure (hPa)	Maximum wind speed (m/s)	Concentration (copies/m^3^)	Positive rate %
TALIM	06/19∼06/21	985	25	N.D.^a^	0% (0/3)
DOKSURI	06/28∼06/29	995	23	-	-
SAOLA	07/30∼08/03	960	38	N.D.∼25.2	20% (1/5)
HAIKUI	08/06∼08/07	960	35	N.D.∼87.2	50% (1/2)
KAI-TAK	08/14∼08/15	995	20	142.2∼257.5	100% (2/2)
TEMBIN	08/21∼08/28	945	45	N.D.∼126.1	80% (4/5)
JELAWAT	09/27∼09/28	910	55	N.D.	0% (0/2)

a **N.D. means not detected.**

**Figure 4:**
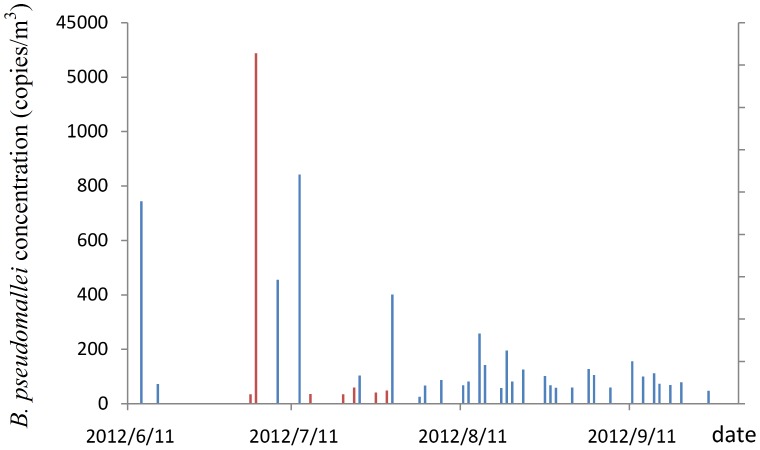
The ambient *B. pseudomallei* concentration profile from June to September 2012.

Table 2 shows the descriptive statistics for ambient *B. pseudomallei* and environmental factors during the typhoon season and reference season. Our results show that ambient *B. pseudomallei*, rainfall, wind, and UV during typhoon season were higher than that during reference season with a significant difference (*p*-value<0.05). However, the concentrations of ambient fungi, O_3_, SO_2_, CO, and NOx were significantly higher during the reference season than typhoon season. Table 3 shows the descriptive statistics for ambient *B. pseudomallei* and environmental factors in June, July, August and September, separately. Figure 4 shows the ambient *B. pseudomallei* concentration profile from June to September.

**Table 2:** Descriptive statistics for ambient *B. pseudomallei* and environmental factors during the typhoon season (June to September) and the reference season (October to December) near Lotus Pond in South Taiwan.

		Typhoon season (June–September)	Reference season (October–December)	*p*-value
*B. pseudomallei* (%)	Positive rate	35.9% (38/106)	0% (0/82)	
	Inhibitory effect	63.2% (24/38)	0%	
*B. pseudomallei* (copies/m^3^)	Mean ± SE (Range)	500.6±3965.4 (N.D.^a^-41376.0)	N.D. (N.D.)	<0.001*
Rainfall (mm)	Mean ± SE (Range)	13.7±31.9 (N.D.-155.0)	0.7±4.5 (N.D.-39.5)	<0.001*
Wind (m/s)	Mean ± SE (Range)	2.1±0.9 (1.1–7.2)	1.8±0.5 (1.1–3.6)	0.029*
UV Index^b^	Mean ± SE (Range)	8.5±2.9 (1.0–13.0)	5.5±1.4 (1.0–8.0)	<0.001*
Ambient Fungi (CFU/m^3^)	Mean ± SE (Range)	400.3±781.1(51.3–2590.4)	869.0±1399.3 (42.9–457.3)	<0.001*
Ambient Bacteria (CFU/m^3^)	Mean ± SE (Range)	241.1±35.4 (68.9–6849.8)	157.9±9.8 (122.5–7884.0)	0.357
O_3_ (ppb)	Mean ± SE (Range)	26.6±1.5 (9.8–72.2)	37.4±1.8 (3.7–75.4)	<0.001*
SO_2_ (ppb)	Mean ± SE (Range)	4.1±0.2 (1.2–10.9)	4.9±0,1 (2.5–9.4)	<0.001*
CO (ppm)	Mean ± SE (Range)	0.4±0.0 (0.1–1.0)	0.6±0.0 (0.3–1.4)	<0.001*
NO_x_ (ppb)	Mean ± SE (Range)	16.6±0.7 (5.1–41.3)	24.2±0.9 (12.4–63.8)	<0.001*

a **N.D. means not detected;**

b **1 unit equals 25 mW/m^2^;**

*significant at ***p*** **-value<0.05.**

**Table 3:** Descriptive statistics for ambient *B. pseudomallei* and environmental factors in June, July, August and September.

		June	July	August	September
*B. pseudomallei* (%)	Positive rate	11.8% (2/17)	35.5% (11/31)	58.1% (18/31)	33.3% (10/30)
*B. pseudomallei* (copies/m^3^)	Range	N.D.^a^-743.0	N.D.-41376.0	N.D.-257.5	N.D.-155.8
	Mean ± SE	47.9±43.7	1656.4±1327.8	47.7±11.3	30.9±8.8
Rainfall (mm)	Range	N.D.-152.5	N.D.-12.5	N.D.-155.0	N.D.-33.0
	Mean ± SE	37.0±11.4	1.7±0.6	24.4±7.5	1.8±1.2
Wind (m/s)	Range	1.8–5.0	1.2–2.9	1.1–7.2	1.3–4.8
	Mean ± SE	2.7±0.2	1.8±0.1	2.4±0.2	1.9±0.1
UV Index^b^	Range	1.0–13.0	4.0–13.0	2.0–11.0	3.0–10.0
	Mean ± SE	7.6±1.1	9.9±0.5	7.8±0.5	8.3±0.3

a **N.D. means not detected;**

b **1 unit equals 25 mW/m^2^.**

### Ambient *B. pseudomallei* and environmental parameters

During June to December, the concentrations of ambient *B. pseudomallei* were positively associated with rainfall and UV of lag 0, lag 1, lag 2, 2-day sum, 3-day sum, and 4-day sum (Table 4). However, no significant correlation was observed between ambient *B. pseudomallei* and environmental parameters during typhoon season. In August, there were four typhoons affecting Taiwan; therefore, we defined August as “typhoon month.” During typhoon month, the concentrations of ambient *B. pseudomallei* were negatively associated with wind speed at lag 0, 2-day sum, and 3-day sum with marginal p- values of 0.079, 0.093 and 0.080, respectively. When we divide environmental parameters by the mean and median values of samples, we found that ambient *B. pseudomallei* of rainy days (>median value of 0 mm) was significantly higher than that of no rainfall days with *p*-value of 0.006 during June to December (Table 5). During typhoon month, ambient *B. pseudomallei* of high-wind-speed days divided by mean value (2.4 m/s) were significantly lower than that of low-wind-speed days.

**Table 4:** The association between ambient *B. pseudomallei* and environmental parameters of lag 0, lag 1, lag 2, 2-days sum, 3-days sum, and 4-days sum during whole period (June to December), typhoon season (June to September), and typhoon month (August).

Ambient *B. pseudomallei* (copies/m^3^)							
		Whole period (June–December) (N = 191)		Typhoon season (June to September) (N = 109)		Typhoon month (August) (N = 31)	
		r	*p*-value	r	*p*-value	r	*p*-value
Rainfall	lag 0	0.184	0.011*	−0.041	0.671	−0.050	0.788
	lag 1	0.140	0.053	−0.074	0.446	−0.140	0.453
	lag 2	0.176	0.015*	−0.044	0.653	−0.052	0.779
	lag 3	0.220	0.002*	0.012	0.899	0.255	0.166
	2-days sum	0.181	0.012*	−0.065	0.500	−0.115	0.537
	3-days sum	0.218	0.002*	−0.038	0.694	−0.135	0.468
	4-days sum	0.235	0.001*	−0.028	0.773	−0.039	0.834
Wind Speed	lag 0	−0.019	0.794	−0.155	0.108	−0.320	0.079
	lag 1	0.004	0.952	−0.133	0.168	−0.032	0.863
	lag 2	0.102	0.161	0.014	0.888	−0.066	0.722
	lag 3	0.137	0.059	0.103	0.285	0.266	0.148
	2-days sum	−0.014	0.848	−0.176	0.067	−0.307	0.093
	3-days sum	0.034	0.642	−0.139	0.150	−0.319	0.080
	4-days sum	0.086	0.237	−0.074	0.442	−0.197	0.287
UV	lag 0	0.246	0.001*	0.021	0.832	0.045	0.809
	lag 1	0.215	0.003*	−0.027	0.777	0.126	0.500
	lag 2	0.202	0.005*	−0.035	0.721	0.159	0.392
	lag 3	0.298	<0.001*	0.139	0.150	0.020	0.913
	2-days sum	0.263	<0.001*	−0.008	0.936	0.090	0.632
	3-days sum	0.282	<0.001*	−0.017	0.864	0.189	0.310
	4-days sum	0.320	<0.001*	0.050	0.608	0.150	0.420

* ***p*** **-value<0.05;** lag 0, lag 1, lag 2, and lag 3: the effects of 0-, 1-, 2-, 3-day lags before the date ambient *B. pseudomallei* measurement.

**Table 5:** Associations between ambient *B. pseudomallei* concentrations and environmental parameters (rainfall and wind speed) divided by the mean and median values during whole period (June to December), typhoon season (June to September), and typhoon month (August).

	Ambient *B. pseudomallei* (copies/m^3^)											
	whole period (June–December)				Typhoon season (June–September)				Typhoon month (August)			
		N	mean	*p*-value		N	mean	*p*-value		N	mean	*p*-value
Divided by the mean value of samples	Rainfall				Rainfall				Rainfall ≧			
	≧8.1 mm	26	45.0	0.17	≧13.7 mm	22	49.7	0.49	24.4 mm	10	33.3	0.85
	<8.1 mm	165	325.3		<13.7 mm	87	621.2		<24.4 mm	21	54.5	
	Wind speed				Wind				Wind			
	≧2.0 m/s	65	680.6	0.91	≧2.1 m/s	36	1153.6	0.16	≧2.4 m/s	9	22.5	<0.001*
	<2.0 m/s	126	77.2		<2.1 m/s	73	165.1		<2.4 m/s	22	58.0	
Divided by the median value of samples	Rainfall				Rainfall				Rainfall			
	>0.0 mm	69	101.5	0.006*	>0.1 mm	54	120.7	0.42	>1.5 mm	14	32.7	0.60
	≦0.0 mm	122	389.9		≦0.1 mm	55	873.7		≦1.5 mm	17	60.0	
	Wind speed				Wind				Wind			
	>1.8 m/s	94	493.7	0.80	>1.9 m/s	54	836.0	0.10	>2.0 m/s	15	39.7	0.36
	≦1.8 m/s	97	84.2		≦1.9 m/s	55	171.4		≦2.0 m/s	16	55.2	

## Discussion

In this study, we developed a filtration/real-time qPCR method to quantify ambient *B. pseudomallei* with a wide dynamic range over 8 orders of magnitude and a correlation coefficient (r) value of 0.998. The detection limit was 6.94 copy/m^3^, which is much lower than that in the previous studies for airborne *M. tuberculosis* (583 copy/m^3^) and airborne influenza A, B, and A/H5 virus (886, 653 and 1236 copy/m^3^, respectively) [26]–[27]. The low detection limit and wide linear range demonstrate that this newly established method is a promising tool for deeper insight into transmissibility and epidemiology of melioidosis, as well as infection control.

In our laboratory evaluation, Teflon filters show better performance when compared to PC filters. Teflon filters were also used for measuring airborne influenza virus in poultry markets and ambient influenza virus [27]–[28]. Our results showed that samples transported at 4°C were superior to 25°C. Inhibitory effects were also observed. In a previous study, authors found that air samples containing bacteria and fungi may inhibit PCR amplification and dilution of these samples can resolve these problems [34]. Therefore, optimistic protocols for ambient *B. pseudomallei* quantification in our follow-up field evaluations used Teflon filters to sample ambient *B. pseudomallei*, transported samples at 4°C to our laboratory within one hour, and analyzed samples simultaneously using 1, 1/10 and 1/100 dilutions.

Melioidosis is a severe bacterial infection with high case-fatality rates of approximately 40% in Thailand, 30% to 14% in Australia, 40% in Singapore, and 22.7% in Taiwan [1], [6], [9]–[11]. It is now believed that percutaneous inoculation is the major mode of acquisition due to exposure history to polluted water and mud, high risk among farmers, and wide isolation of *B. pseudomallei* from soil, mud, and pooled surface water in endemic areas [1], [19]. However, pneumonia was the most common clinical presentation, which accounted for 32.6%, 51%, 45%, 42.1% and 70% of cases in India [5], northern Australia [10], Thailand [1], Malaysia [15], and Taiwan [14], respectively. In addition, epidemiological studies hypothesized that inhalation of *B. pseudomallei* was the mode of transmission after heavy monsoonal rains and winds [5], [6], [10], [13], [17], [20]–[22]. In this study, we successfully quantified ambient *B. pseudomallei* using filtration/real-time qPCR. To our knowledge, this is the first report describing concentrations of *B. pseudomallei* in ambient air. Our results provided evidence to support the hypothesis from these epidemiological studies of inhalation transmission.

*B. pseudomallei* is widely isolated from soil and water samples [35]–[38]. In Taiwan, there was a cluster of melioidosis for both cases and deaths in the Zoynan Region [11]. *B. pseudomallei* was isolated from this region at a rate of 25.9% and 13.5% for soil samples and water samples, respectively [11]. Our results of 20.2% positive air samples was similar to that of water and soil samples and implicated that ambient *B. pseudomallei* should be a concern as an important source of transmission.

In 2012, seven typhoons affected Taiwan whereas only three typhoons (Doksuri, Kai-Tak, and Tembin) affected Zoynan Region. We lost the ambient *B. pseudomallei* samples during Doksuri-affected periods due to communication mistakes with the personnel managing the sampling site. For the other two typhoons affecting the Zoynan Region, the positive rates of ambient *B. pseudomallei* were very high at 100% and 80% for Kai-Tak and Tembin, respectively. In addition, ambient *B. pseudomallei* were only detected during the typhoon season when comparing to the reference season. Our results support the hypothesis that heavy monsoonal rains and winds may cause a shift toward inhalation of *B. pseudomallei* [13] and provide a possible explanation for the observed growth in the number of melioidosis cases in the Taiwan Zoynan Region from the 2005 and 2009 typhoon season [16], [20]. Regarding air pollutants, the significantly higher concentrations of ambient fungi, O_3_, SO_2_, CO and NO were observed during the reference season when compared to the typhoon season. Pollutants concentrated in autumn due to high atmospheric pressure may provide the explanation [39].

When investigating ambient *B. pseudomallei* in June, July, August and September separately, we found that although the highest positive rate was observed in August, the highest concentrations were all observed in July with the lowest rainfall among June to September (Table 3, Figure 4). This observation is contrary to the results in the previous studies [5]–[6], [10], [13]–[14]. During our sampling period, we found that the water of the Lotus Pond was pumped out on June 27 to let sediment expose to the air. The authority (Department of Health, Kaohsiung city government) did confirm that water was pumped out on June 27 prior to the typhoon to avoid waterlogging. Since we lost samples from June 28 to June 30, we compared the concentration of June and July to see the association between ambient *B. pseudomallei* and the appearance of sediment. The concentration of ambient *B. pseudomallei* in July was higher than that in June with a marginal *p*-value of 0.052. For evaluating the association between the appearance of sediment and ambient *B. pseudomallei* from June to September, the linear-by-linear association test was used after logarithmic transformation of ambient *B. pseudomallei*. We defined log (ambient *B. pseudomallei* +1) as the dependent variable (Y) and the time period after the appearance of sediment was grouped by two weeks as X = 6, 5, 4, 3, 2, 1 for data between July 1 to July 15, July 16 to July 31, August 1 to August 15, August 16 to August 31, September 1 to September 15, September 16 to September 30, respectively, where the data before the appearance of sediment was defined as X = 0. The sediment “event” was significantly associated with ambient *B. pseudomallei* with the predicted model as Y = 0.459+0.123×(*p*-value 0.024). Our results show that the appearance of sediment at a nearby pond may be the source of ambient *B. pseudomallei* during the typhoon season.

The association between melioidosis and rainfall intensity is well documented from endemic regions, with 75%, 72%, 80%, 81% and 85% of cases presenting during the wet season in northeast Thailand [6], India [5], Taiwan [14], northern Australia [10] and northern Australia [13], respectively. In regard to the associations between ambient *B. pseudomallei* and environmental parameters, we found that rainfall was positively correlated to ambient *B. pseudomallei* during June to December. This is consistent with the previous observation of significantly linear correlation between melioidosis cases and rainfall [5]–[6], [13], [15]–[16]. However, no significant correlation was observed between ambient *B. pseudomallei* and rainfall during the typhoon season and typhoon month. This may be due to the appearance of sediment strongly affecting the concentration of ambient *B. pseudomallei*. We defined August as “typhoon month” to investigate the association between ambient *B. pseudomallei* and environmental parameters due to four typhoons affecting Taiwan in August, and less effect of sediment appearance in August when compared to July. We defined log (ambient *B. pseudomallei* +1) as the dependent variable (Y). Rainfall, wind speed and typhoon were defined as independent variables. We found that typhoon and wind speed were significantly associated with ambient *B. pseudomallei* with the predicted model as Y = 1.350-0.272 (wind speed) (*p*-value 0.039)+0.713 (typhoon) (*p*-value 0.046) with a *p*-value of 0.049 for the whole model. We found that typhoon was positively correlated with ambient *B. pseudomallei*. This supports conclusions from previous studies that the case clusters were associated with sudden and heavy rainfall related to cyclones and typhoons [1], [4], [6], [10], [14]–[18], [22]. Wind speed was reversely correlated with ambient *B. pseudomallei*. This may be due to dilution of ambient *B. pseudomallei* by wind.

In conclusion, we successfully developed a filtration/real-time qPCR method to quantify ambient *B. pseudomallei*. To our knowledge, this is the first report describing concentrations of ambient *B. pseudomallei*. Our results provided evidence to support the hypothesis that heavy monsoonal rains and winds may cause a shift toward inhalation of *B. pseudomallei*. Ambient *B. pseudomallei* should be concerned as an important source of transmission due to the wind speed during the typhoon season. Ambient *B. pseudomallei* should be taken seriously as a surveillance target in endemic areas of Melioidosis especially during typhoon season.
